# Weighted Epworth sleepiness scale predicted the apnea-hypopnea index better

**DOI:** 10.1186/s12931-020-01417-w

**Published:** 2020-06-12

**Authors:** Qi Guo, Wei-dong Song, Wei Li, Chao Zeng, Yan-hong Li, Jian-ming Mo, Zhong-dong Lü, Mei Jiang

**Affiliations:** 1 Department of Pulmonary and Critical Care Medicine, Shenzhen Hospital, Peking University, Lianhua road No. 1120, Shenzhen, 518036 Guangdong China; 2 State Key Laboratory of Respiratory Disease, National Clinical Research Center for Respiratory Disease, Guangzhou Institute of Respiratory Health, The First Affiliated Hospital, Guangzhou Medical University, Guangzhou, 510120 Guangdong China

**Keywords:** Obstructive sleep apnea syndrome, Epworth sleepiness scale, Weighted Epworth sleepiness scale, Apnea-hypopnea index, Priority

## Abstract

**Background:**

The relationship between the Epworth sleepiness scale (ESS) and the apnea-hypopnea index (AHI) is uncertain and even poor. The major problem associated with the ESS might be a lack of consideration of weight in prediction in clinical practice. Would awarding different item-scores to the four scales of ESS items to develop a weighted ESS scoring system improve the accuracy of the AHI prediction? It is warranted to explore the intriguing hypotheses.

**Methods:**

Seven hundred fifty-six adult patients with suspicion of obstructive sleep apnoea syndrome (OSAS) were prospectively recruited to a derivation cohort. This was tested against a prospective validation cohort of 810 adult patients with suspected OSAS. Each ESS item’s increased odds ratio for the corresponding AHI was calculated using univariate logistic regression. The receiver operating characteristic curves were created and the areas under the curves (AUCs) were calculated to illustrate and compare the accuracy of the indices.

**Results:**

The higher the ESS item-score, the closer the relationship with the corresponding AHI. The odds ratios decreased as a result of the increased AHI. The ESS items were of unequal weight in predicting the corresponding AHI and a weighted ESS was developed. The coincidence rates with the corresponding AHI, body mass indices, and neck circumferences rose as the scores increased, whereas nocturnal nadir oxygen saturations decreased, and the weighted ESS was more strongly associated with these indices, compared with the ESS. The capability in predicting the patients without OSAS or with severe OSAS was strong, especially the latter, and the weighted ESS orchestrated manifest improvement in screening the patients with simple snoring. The patterns of sensitivities, specificities, and Youden’s indices of the four ranks of weighted ESS for predicting the corresponding AHI were better than those of the ESS, and the AUCs of weighted ESS were greater than the corresponding areas of ESS in the two cohorts.

**Conclusions:**

The weighted ESS orchestrated significant improvement in predicting the AHI, indicating that the capability in predicting the patients without OSAS or with severe OSAS was strong, which might have implications for clinical triage decisions to prioritize patients for polysomnography.

## Introduction

Obstructive sleep apnoea (OSA) is a major challenge for physicians and healthcare systems throughout the world [1]. OSA is characterised by repeated interruption of breathing during sleep due to episodic collapse of the pharyngeal airway, nocturnal hypoxaemia and sleep fragmentation. This sleep disruption commonly causes excessive daytime sleepiness (EDS) [2]. Nocturnal hypoxaemia can be a major determinant of EDS in patients with obstructive sleep apnoea syndrome (OSAS) [3]. The apnea-hypopnea index (AHI) is an objective, sensitive and specific measure of the severity of OSA [4]. The extensively validated Epworth sleepiness scale (ESS), which is a brief self-administered questionnaire that asks the subject to rate on a scale of 0–3 the chances that he would have dozed in eight most soporific situations commonly met in daily life, is the most frequently used instrument for assessing subjective daytime sleepiness or sleep propensity in adults [5, 6]. The external criterion validity of the ESS has been tested by examining the relationship between ESS scores and the AHI, but unfortunately, the relationship is uncertain and even poor [1, 3, 5, 7–9].

It is self-evident that we are more likely to fall asleep when lying down than when standing up. Sleep propensity must also be distinguished from the state and process of fatigue [10]. The ESS tries to overcome the fact that people have different daily routines, some facilitating and others inhibiting daytime sleep [5]. Hence, the ESS was designed to measure daytime sleepiness over the whole range, from very high to low levels. The items were chosen, therefore, to represent situations of a widely differing soporific nature, some known to be very soporific; others less so [6]. Johns stated that a corollary of his model of sleep and wakefulness is that some postures, activities and environmental situations will be more conducive than others to sleep-onset and created the term somnificity characterizing a posture, activity and environmental situation that reflects its capacity to facilitate sleep-onset in a majority of subjects to replace the phrase, soporific nature of a situation [10]. However, the major problem associated with the ESS in uncertain and even poor prediction of the AHI might be a lack of consideration of weight in clinical practice. Would awarding different item-scores to the four scales of ESS items to develop a weighted ESS scoring system improve the accuracy of the AHI prediction? Therefore, it is warranted to explore the intriguing hypotheses.

Two prospective cohort studies were conducted to derive and validate a weighted ESS.

## Materials and methods

### Design and setting

A prospective derivation cohort study of 756 adult patients with suspicion of OSAS was conducted at the Department of Pulmonary and Critical Care Medicine in a 1600-bed tertiary care university hospital from June 2018, through November 2018. We then performed a prospective validation cohort study of 810 adult patients with suspected OSAS who presented to our hospital between December 2018 and June 2019.

### Criteria for enrollment

International Classification of Sleep Disorders (ICSD) diagnostic criteria for OSA were referred [11, 12].

Clinical suspicion of OSAS was based on complaints of (1) loud snoring or witnessed apneas, (2) EDS, or (3) overweight/obesity, which was reported by the patient or relatives. The exclusion criteria were heart failure, dementia, major psychiatric disorder, or another condition not suitable for the use of polysomnography.

### Polysomnography

In accordance with standard techniques [13, 14], a computer data acquisition and analysis system recorded the following signals: electroencephalogram, bilateral electrooculogram, electrocardiogram, submental and bilateral anterior tibialis electromylogram, thoracic and abdominal excursion, oral and nasal airflow by thermistor and breath sounds, body position, and oxygen saturation by pulse oximeter.

### Outcome

The main outcome measures were the ESS, weighted ESS scores and the AHI. Secondary outcomes incorporated body mass index (BMI), neck circumference (NC), and lowest oxygen saturation (LOS).

### Sample size calculation

Unit-level design prevalence, cluster-level design prevalence, test sensitivity, target cluster sensitivity, and target system sensitivity were 20%, 1%, 0.9, 0.5, and 0.95, respectively. The total numbers of clusters to be sampled were 598, and the maximum number of samples was 2392.

### Data collection

Seven hundred fifty-six patients were enrolled into the derivation cohort excluding 9 cases due to exclusion criteria and 810 into the validation cohort excluding 11 cases. Overnight polysomnography was arranged for all patients with suspected OSAS. Clinical and diagnostic data were collected. The ESS, weighted ESS scores and BMI were calculated. The statistician was blinded to the study.

### Statistical analysis

All statistical analyses were performed with Statistical Package for the Social Science for Windows version 16.0 (SPSS, Chicago, IL, USA) and MedCalc version 19.1 (Mariakerke, Belgium). Categorical variables and continuous variables were reported as the percentages and the mean ± standard deviation (SD), respectively. Chi-Square test, unpaired Student’s t-test, one-way ANOVA, univariate logistic regression, and Spearman rank correlation were employed. Two groups were compared by unpaired Student’s t-test or Chi-Square test, and analyses of multiple groups were carried out using one-way ANOVA or Chi-Square test, depending on the characteristics of variables. Each ESS item’s increased odds ratio (OR) for the corresponding AHI was calculated using univariate logistic regression. The receiver operating characteristic (ROC) curves were created and the areas under the ROC curves (AUCs) were calculated to illustrate and compare the accuracies of the indices. The sensitivities, specificities, positive likelihood ratio (PLR), negative likelihood ratio (NLR), positive predictive values (PPVs), negative predictive values (NPVs), and Youden’s indices were also calculated. A *p* value of < 0.05 was considered statistically significant.

## Results

### Baseline characteristics of study cohorts

The demographic and clinical characteristics of recruited patients with suspicion of OSAS were listed in Table 1. The numbers of patients with simple snoring were 116 and 88 in the derivation and validation cohorts, respectively. The participants recruited to the validation cohort were younger and presented higher AHI (especially AHI > 15 and AHI > 30) and lower nocturnal oxygen saturation nadir, compared with those in the derivation cohort.

**Table 1:** Baseline characteristics of study cohorts (Mean ± SD)

Characteristic	Derivation cohort (*n* = 756)	Validation cohort (*n* = 810)	*t or x*^*2*^ value	*p* value
Age (yrs)	43.5 ± 13.0	39.6 ± 12.5	5.579	< 0.001
Male sex (No.) (%)	652 (86.2)	674 (83.2)	2.700	0.100
BMI	26.3 ± 4.2	26.4 ± 4.8	0.679	0.497
NC (cm)	37.9 ± 3.0	37.8 ± 3.4	0.635	0.526
AHI	26.4 ± 20.0	33.8 ± 21.0		
AHI ≥ 5 (No.) (%)	640 (84.7)	722 (89.1)	6.926	0.008
AHI > 15 (No.) (%)	490 (64.8)	670 (82.7)	65.250	< 0.001
AHI > 30 (No.) (%)	290 (38.4)	414 (51.1)	25.694	< 0.001
LOS	79.3 ± 10.1	77.4 ± 9.5	3.926	< 0.001

NOTE: *BMI* Body mass index. *NC* Neck circumference. *ESS* Epworth sleepiness scale. *AHI* Apnea-hypopnea index. *LOS* Lowest oxygen saturation

### Association of the predictive rule of ESS with the AHI in the derivation cohort

In general, the proportions of patients fulfilling the corresponding AHI rose when the ESS item-scores increased. Therefore, the higher the ESS item-score, the closer the relationship with the corresponding AHI (Table 2). However, as the AHI increased, the relationship between the ESS item-score and the corresponding AHI became less close. Each item had a significant increased OR for the corresponding AHI, except for those for AHI > 30 in four items. The ORs decreased as a result of the increased AHI. The top three ORs for AHI ≥ 5 were derived from the items “In a car, while stopped for a few minutes in traffic”, “Sitting and talking to someone”, and “Sitting inactive in a public place (e.g., a theater or a meeting)”, respectively.

**Table 2:** Association of the predictive rule of ESS with the AHI in the derivation cohort (*n* = 756)

Criteria/item score		AHI ≥ 5		*x*^*2*^ value	*p* value	OR (95% CI)	*p* value	AHI > 15		*x*^*2*^ value	*p* value	OR (95% CI)	*p* value	AHI > 30		*x*^*2*^ value	*p* value	OR (95% CI)	*p* value
		Yes	No					Yes	No					Yes	No				
Sitting and reading	0	198	64	71.074	< 0.001	4.877 (3.153-7.543)	< 0.001	196	66	32.317	< 0.001	1.200 (1.015-1.303)	< 0.001	130	132	20.070	< 0.001	1.009 (0.946-1.076)	0.793
	1	276	24					236	64					116	184				
	2	145	0					137	8					87	58				
	3	49	0					47	2					27	22				
Watching TV	0	205	58	50.811	< 0.001	3.793 (2.523-5.702)	< 0.001	193	70	24.151	< 0.001	1.819 (1.410-2.348)	< 0.001	117	146	8.162	0.043	1.246 (1.053-1.476)	0.011
	1	306	30					278	58					154	182				
	2	112	0					104	8					60	52				
	3	46	0					41	4					29	16				
Sitting inactive in a public place (e.g., a theater or a meeting)	0	224	70	71.054	< 0.001	5.199 (3.241-8.340)	< 0.001	208	86	47.677	< 0.001	2.632 (1.969-3.520)	< 0.001	124	170	25.916	< 0.001	1.487 (1.250-1.768)	< 0.001
	1	284	16					252	48					132	168				
	2	125	2					121	6					77	50				
	3	35	0					35	0					27	8				
As a passenger in a car for an hour without a break	0	164	40	38.853	< 0.001	2.316 (1.734-3.095)	< 0.001	148	56	24.843	< 0.001	1.663 (1.347-2.054)	< 0.001	90	114	2.140	0.544	1.057 (0.912-1.229)	0.459
	1	233	40					217	56					137	136				
	2	186	8					174	20					90	104				
	3	85	0					77	8					43	42				
Lying down to rest in the afternoon when circumstances permit	0	81	20	11.846	0.008	1.377 (1.103-1.717)	0.005	81	20	6.991	0.072	1.208 (1.006-1.449)	0.043	51	50	11.919	0.008	1.108 (0.960-1.279)	0.159
	1	170	28					150	48					88	110				
	2	233	20					211	42					105	148				
	3	184	20					174	30					116	88				
Sitting and talking to someone	0	456	82	24.166	< 0.001	5.285 (2.399-11.645)	< 0.001	410	128	36.123	< 0.001	4.542 (2.580-7.996)	< 0.001	238	300	11.438	0.010	1.506 (1.226-1.849)	< 0.001
	1	150	6					144	12					82	74				
	2	48	0					48	0					30	18				
	3	14	0					14	0					10	4				
Sitting quietly after a lunch without alcohol	0	147	46	42.527	< 0.001	2.159 (1.630-2.861)	< 0.001	145	48	8.316	0.040	1.226 (1.004-1.496)	0.045	83	110	5.925	0.115	1.031 (0.886-1.200)	0.691
	1	244	28					230	42					144	128				
	2	207	8					175	40					95	120				
	3	70	6					66	10					38	38				
In a car, while stopped for a few minutes in traffic	0	352	82	52.647	< 0.001	9.019 (4.023-20.218)	< 0.001	314	120	58.246	< 0.001	4.198 (2.724-6.470)	< 0.001	176	258	34.652	< 0.001	1.730 (1.427-2.096)	< 0.001
	1	212	6					200	18					112	106				
	2	82	0					80	2					52	30				
	3	22	0					22	0					20	2				

NOTE: *ESS* Epworth sleepiness scale. *AHI* Apnea-hypopnea index. *OR* Odds ratio. *CI* Confidence interval

### Derivation of the weighted ESS

The higher the ESS score, the higher the person’s average sleep propensity in daily life, according to high ESS scores indicative of EDS in patients with OSAS. On the basis of the weight of predictive rules of ESS for OSA in predicting the AHI, the eight ESS items were divided into five ranks and different item-scores were assigned for different ranks to develop a weighted ESS scoring system. 0–5–6-7 item-scores were assigned for the four scales of three ESS items with the top three ORs, except for the item “Sitting inactive in a public place (e.g., a theater or a meeting)” due to its lower OR for AHI > 15, compared with the other two items. The other item-scores (0–4–5-6 for one item, 0–3–4-5 for two items, 0–2–3-4 for one item, and 0–1–2-3 for two items) were shown in Table 3. All item-scores were intended to be integers. These scores would be taken at face value if some people could not decide on one number and reported half-values. It would be rounded up to the next whole number if the total score included a half-value after adding them up. The ESS score (the sum of eight item-scores, 0–3) ranged from 0 to 24, and the weighted ESS score (the sum of eight item-scores, from 0 to 3 to 0–7) from 0 to 40.

**Table 3:** The ESS and weighted ESS scoring systems

Variable	ESS				Weighted ESS			
	I	II	III	IV	I	II	III	IV
Sitting and reading	0	1	2	3	0	3	4	5
Watching TV	0	1	2	3	0	3	4	5
Sitting inactive in a public place (e.g., a theater or a meeting)	0	1	2	3	0	4	5	6
As a passenger in a car for an hour without a break	0	1	2	3	0	2	3	4
Lying down to rest in the afternoon when circumstances permit	0	1	2	3	0	1	2	3
Sitting and talking to someone	0	1	2	3	0	5	6	7
Sitting quietly after a lunch without alcohol	0	1	2	3	0	1	2	3
In a car, while stopped for a few minutes in traffic	0	1	2	3	0	5	6	7
Total scores	0–24				0–40			

NOTE: *ESS* Epworth sleepiness scale. I: Would never doze. II: Slight chance of dozing. III: Moderate chance of dozing. IV: High chance of dozing

### Associations of AHI, BMI, NC and LOS with the ESS and weighted ESS scores

In general ESS scores can be interpreted as follows: 0–10 indicates normal daytime sleepiness (NDS), 11–12 mild EDS, 13–15 moderate EDS, and 16–24 severe EDS. Similarly, 0–14 rank in the weighted ESS scores was defined as NDS, and the other ranks were described in Table 4. The four ranks of ESS scores were regarded as corresponding with AHI < 5, AHI ≥ 5, AHI > 15, and AHI > 30, respectively. As did the four ranks of weighted ESS scores. In general, the coincidence rates with the corresponding AHI rose sharply as the cut-off values of scores increased in the two scoring systems in the two cohorts, and the weighted ESS was more strongly associated with the corresponding AHI, especially in the rank for NDS, compared with the ESS.

**Table 4:** Relationships between the ESS and weighted ESS scores and the AHI

Features	Cut-off values	Derivation cohort (*n* = 756)		Validation cohort (*n* = 810)	
		Total	Coincidence with corresponding AHI (%)	Total	Coincidence with corresponding AHI (%)
ESS					
NDS	0–10	354	108 (30.5)	438	88 (20.1)
Mild EDS	11–12	136	44 (32.4)	92	8 (8.7)
Moderate EDS	13–15	110	68 (61.8)	96	32 (33.3)
Severe EDS	16–24	156	152 (97.4)	184	178 (96.7)
Weighted ESS					
NDS	0–14	124	100 (80.6)	196	88 (44.9)
Mild EDS	15–19	194	104 (53.6)	122	48 (39.3)
Moderate EDS	20–29	252	142 (56.3)	242	152 (62.8)
Severe EDS	30–40	186	184 (98.9)	250	242 (96.8)

NOTE: *ESS* Epworth sleepiness scale. *AHI* Apnea-hypopnea index. *NDS* Normal daytime sleepiness. *EDS* Excessive daytime sleepiness

BMI and NC increased significantly as a result of the increased ranks in the two scoring systems in the two cohorts, whereas LOS decreased (Table 5). The weighted ESS was more strongly associated with these indices in the two cohorts, compared with the ESS.

**Table 5:** Associations of BMI, NC and LOS with the ESS and weighted ESS scores (Mean ± SD. Derivation cohort, *n* = 756; validation cohort, *n* = 810)

Scores	BMI		NC (cm)		LOS (%)	
	Derivation cohort	Validation cohort	Derivation cohort	Validation cohort	Derivation cohort	Validation cohort
ESS						
0–10	25.50 ± 4.35	25.53 ± 4.19	37.09 ± 3.14	37.19 ± 3.07	83.28 ± 8.61	79.62 ± 8.68
11–12	26.48 ± 4.50	26.00 ± 4.12	37.76 ± 2.91	38.15 ± 3.37	78.49 ± 9.91	76.65 ± 10.13
13–15	25.99 ± 3.04	26.82 ± 6.51	38.69 ± 2.25	37.89 ± 4.03	77.31 ± 7.46	76.44 ± 8.17
16–24	27.94 ± 3.73	28.49 ± 4.81	39.17 ± 2.63	38.88 ± 3.68	72.55 ± 11.05	72.95 ± 9.94
*F* value	13.161	17.837	22.338	11.347	51.503	23.957
*p* value	< 0.001	< 0.001	< 0.001	< 0.001	< 0.001	< 0.001
*r*_s_ value	0.259	0.248	0.283	0.193	−0.452	−0.294
*p* value	< 0.001	< 0.001	< 0.001	< 0.001	< 0.001	< 0.001
Weighted ESS						
0–14	25.01 ± 5.00	24.91 ± 3.97	35.84 ± 3.38	36.56 ± 3.01	88.48 ± 4.93	81.33 ± 8.48
15–19	25.27 ± 3.82	26.15 ± 4.64	37.52 ± 2.81	37.60 ± 2.91	82.00 ± 7.13	79.05 ± 8.13
20–29	26.57 ± 3.92	26.38 ± 5.12	38.19 ± 2.72	38.15 ± 3.57	77.76 ± 9.74	77.55 ± 9.39
30–40	27.66 ± 3.82	27.73 ± 4.85	39.16 ± 2.51	38.43 ± 3.61	72.58 ± 10.43	73.33 ± 9.36
*F* value	15.572	13.235	36.825	12.762	92.166	30.979
*p* value	< 0.001	< 0.001	< 0.001	< 0.001	< 0.001	< 0.001
*r*_s_ value	0.279	0.244	0.337	0.214	− 0.565	−0.351
*p* value	< 0.001	< 0.001	< 0.001	< 0.001	< 0.001	< 0.001

NOTE: *BMI* Body mass index. *NC* Neck circumference. *LOS* Lowest oxygen saturation. *ESS* Epworth sleepiness scale. *SD* Standard deviation. *CM* Centimeter. *r*_*s*_ Rank correlation coefficient

### Comparisons of the scoring systems for predicting the AHI

The sensitivities, specificities, and predictive values of the different cut-off values in the two scoring systems for predicting the corresponding AHI were shown in Table 6. The two scoring systems demonstrated the highest sensitivities, NPVs and Youden’s indices, and the lowest NLRs in the lowest rank, and the highest specificities, PLRs and PPVs, and higher Youden’s indices in the highest rank in the two cohorts, and the patterns of sensitivities, specificities, and Youden’s indices of the ranks for NDS and severe EDS for prediction of the corresponding AHI were better than those of the other two intermediate ranks in the two cohorts, except for that of the rank for mild EDS in the weighted ESS in the validation cohort, indicating that the capability in predicting the patients without OSAS or with severe OSAS was strong, especially the latter, and that the weighted ESS orchestrated manifest improvement in screening the patients with simple snoring. The patterns of sensitivities, specificities, and Youden’s indices of the four ranks of weighted ESS for predicting the corresponding AHI were better than those of the ESS in the two cohorts, indicating that the weighted ESS orchestrated significant improvement in predictive power.

**Table 6:** Test characteristics of the ESS and weighted ESS with different cut-off values for the corresponding AHI

Rule	Cut-off values	Sensitivity (%)	Specificity (%)	PLR	NLR	PPV (%)	NPV (%)	Youden’s index
Derivation cohort (*n* = 756)								
ESS								
NDS	0–10	93.1	61.6	2.424	0.112	30.5	98.0	0.55
Mild EDS	11–12	29.3	84.8	1.928	0.834	32.4	82.9	0.14
Moderate EDS	13–15	35.8	92.6	4.838	0.693	61.8	81.1	0.28
Severe EDS	16–24	52.4	99.1	58.222	0.480	97.4	77.0	0.52
Weighted ESS								
NDS	0–14	86.2	96.3	23.298	0.143	80.6	97.5	0.83
Mild EDS	15–19	69.3	85.1	4.651	0.361	53.6	91.8	0.54
Moderate EDS	20–29	71	80.2	3.586	0.362	56.3	88.5	0.51
Severe EDS	30–40	63.4	99.6	158.5	0.367	98.9	81.4	0.60
Validation cohort (*n* = 810)								
ESS								
NDS	0–10	100	51.5	2.062	0	20.1	100	0.52
Mild EDS	11–12	15.4	88.9	1.387	0.952	8.7	93.9	0.04
Moderate EDS	13–15	12.3	88.4	1.06	0.988	33.3	68.1	0.01
Severe EDS	16–24	43.4	98.5	28.933	0.575	96.7	62.9	0.42
Weighted ESS								
NDS	0–14	100	85.0	6.667	0	44.9	100	0.85
Mild EDS	15–19	92.3	90.2	9.418	0.081	39.3	99.4	0.83
Moderate EDS	20–29	58.5	83.6	3.567	0.496	62.8	81.0	0.42
Severe EDS	30–40	59.0	98.0	29.5	0.418	96.8	70.0	0.57

NOTE: *ESS* Epworth sleepiness scale. *AHI* Apnea-hypopnea index. *PLR* Positive likelihood ratio. *NLR* Negative likelihood ratio. *PPV* Positive predictive value. *NPV* Negative predictive value. *NDS* Normal daytime sleepiness. *EDS* Excessive daytime sleepiness

**Fig. 1:**
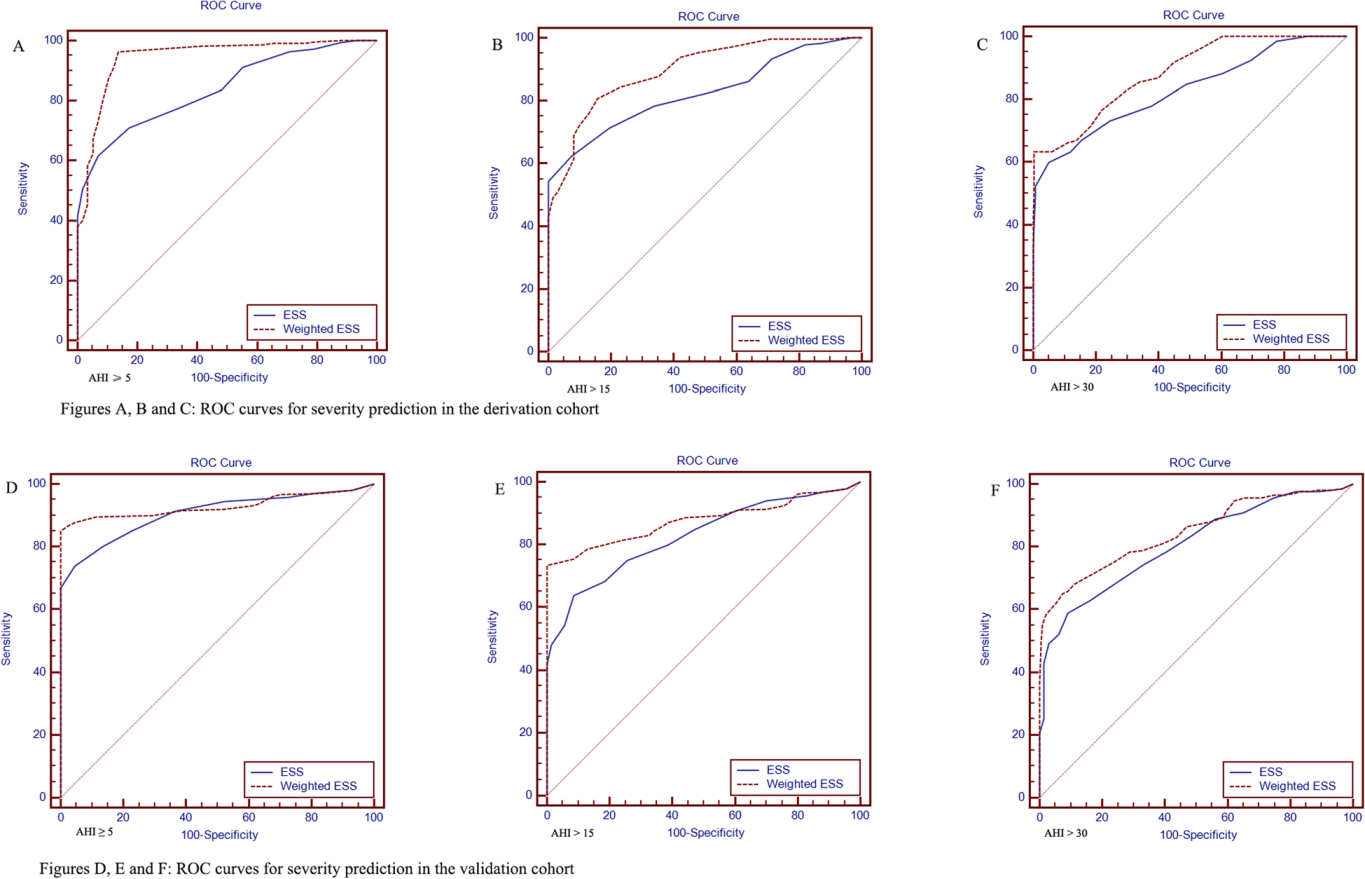
ROC curves for severity prediction. ROC: The receiver operating characteristic. ESS: Epworth sleepiness scale

The ROC curves for the two scoring systems in the two study populations illustrated the differences in accuracy of the AHI prediction (Table 7, and Fig. 1). In general, AUC values decreased as the AHI increased. The weighted ESS was performed better than the ESS in the two cohorts.

**Table 7:** AUC values for the two scoring systems to predict the corresponding AHI and their comparisons

Feature	Derivation cohort (*n* = 756)			Validation cohort (*n* = 810)		
	AUC value	Standard error	95% CI	AUC value	Standard error	95% CI
ESS						
AHI ≥ 5	0.840	0.0160	0.811–0.865	0.906	0.0114	0.884–0.925
AHI > 15	0.825	0.0143	0.797–0.852	0.827	0.0153	0.799–0.852
AHI > 30	0.829	0.0161	0.800–0.855	0.806	0.0151	0.777–0.833
Weighted ESS						
AHI ≥ 5	0.945	0.0122	0.927–0.960	0.930	0.0088	0.910–0.946
AHI > 15	0.897	0.0111	0.873–0.917	0.877	0.0117	0.852–0.899
AHI > 30	0.885	0.0120	0.860–0.906	0.847	0.0136	0.820–0.871
	Difference	z statistic	*p* value	Difference	z statistic	*p* value
AHI ≥ 5 ESS ~ Weighted ESS	0.106	7.230	< 0.0001	0.0237	3.092	0.0020
AHI > 15 ESS ~ Weighted ESS	0.0711	6.519	< 0.0001	0.0498	5.462	< 0.0001
AHI > 30 ESS ~ Weighted ESS	0.0557	6.478	< 0.0001	0.0407	4.166	< 0.0001

NOTE: *AUC* The area under the receiver operating characteristic curve. *AHI* Apnea-hypopnea index. *CI* Confidence interval. *ESS* Epworth sleepiness scale

## Discussion

The main findings of the current study comprise the following: The higher the ESS item-score, the closer the relationship with the corresponding AHI. As the AHI increased, the relationship between the ESS item-score and the corresponding AHI became less close. The ORs decreased as a result of the increased AHI. The ESS items were of unequal weight in predicting the corresponding AHI and a weighted ESS was developed. The coincidence rates with the corresponding AHI, BMIs and NCs rose as the cut-off values of scores increased in the two cohorts, whereas LOS decreased, and the weighted ESS was more strongly associated with these indices, compared with the ESS. The capability in predicting the patients without OSAS or with severe OSAS was strong, especially the latter, and the weighted ESS orchestrated manifest improvement in screening the patients with simple snoring. The patterns of sensitivities, specificities, and Youden’s indices of the four ranks of weighted ESS for predicting the corresponding AHI were better than those of the ESS, and the AUCs of weighted ESS were greater than the corresponding areas of ESS in the two cohorts.

Additional 4 scores was added to the top two ESS items while keeping a “0” unchanged, and so on. In other words, any positive answer to the top two items is clinically more relevant to the AHI than any other positive answer to the other items. Onen and coworkers adopted this weighted scoring strategy to develop a simple three-item instrument for measuring an older patient’s daytime sleepiness duration and general level of sleepiness in daily activities that can also include information obtained from a proxy [15].

Item “Lying down to rest in the afternoon when circumstances permit” is the only one that clearly involves lying down. All other items involve variations of the sitting posture, except item “watching TV” in which the posture is not specified [10]. The situation in the above-mentioned item was the most soporific. By contrast, the situations in items “Sitting and talking to someone” and “In a car, while stopped for a few minutes in traffic” were the least soporific. The other situations in the other items were intermediate in their soporific nature [6]. Furthermore, there were significant overall differences in item-ranks according to their relative somnificities among the eight ESS items, and the items with the top five ranks were “Lying down to rest in the afternoon when circumstances permit”, “Watching TV”, “Sitting and reading”, “As a passenger in a car for an hour without a break”, and “Sitting quietly after a lunch without alcohol”, respectively [10], which demonstrated the least five ORs for the AHI in the current study. Sleep propensity was manifested when lying down. Therefore, the item “Lying down to rest in the afternoon when circumstances permit” demonstrated the lowest OR. On the contrary, sleep propensity was decreased in a car while stopped for a few minutes in traffic, which was the least somniferous item and showed the highest OR. As a result, the somnificities were not paradoxical but concordant in the above-mentioned studies including the current. Therefore, the current findings were enough to be clinically relevant. Doubt should not be cast on this scale of somnificities, which may be widely applicable.

The finding that ESS scores can distinguish patients who simply snore from those with even mild OSAS is evidence for the sensitivity of the ESS and the questionnaire should be useful in elucidating the epidemiology of snoring and OSAS [5]. The capability in screening patients with simple snoring or severe OSAS was strong, especially the latter, and the weighted ESS did better than the ESS in the current study. It might be the causations that the subjective reports on the item-scores when never dozing or having high chance of dozing were relatively more accurate and that the consideration of weight in prediction might embody the true and natural features of ESS items, which might avoid underestimation of some variables. Nocturnal polysomnography is the gold standard for diagnosing OSAS, but the diagnostic procedures are expensive and time-consuming. On the basis of the high prevalence of snoring and OSAS, many sleep laboratories have large numbers of snorers waiting to be tested. The weighted ESS could more accurately detect the patients with simple snoring or severe OSAS, especially the former, owing to higher Youden’s indices, which might have implications for clinical triage decisions to prioritize patients for polysomnography. The patients fulfilling severe EDS would have the priority for polysomnography, whereas those meeting NDS would not. Moreover, the weighted ESS predicted the AHI better and was more strongly linked to BMI, NC and LOS compared with the ESS, indicating that the weighted ESS orchestrated significant improvement in predicting severity in patients with OSAS. These results give credence to the future recommendation for decision making in clinical practice, although much more research is needed to investigate this matter, especially the generalisability.

Practicability is another aspect that requires assessment when developing a new scoring system. In consideration of the ESS relatively easier to practically implement, the weighted ESS is a little more difficult to implement, but the benefit far outweighs the difficulty. Hence, the practicability might not be too bad.

The relationship with the corresponding AHI became closer as a result of the higher item-score. The higher the ESS item-score, the higher the somnificity. This might be the causation resulting in the relationship. The relative inaccuracy of subjective reports on the item-scores might be more obvious when having low chance of dozing than having high chance. It might also be envisaged to interpret the relationship. As the AHI increased, the ESS items demonstrated lower ORs. What mechanisms might be envisaged to interpret this phenomenon? It remains further research.

## Limitations

Several limitations of this study must be acknowledged. First, the relative inaccuracy of subjective reports in the ESS and weighted ESS was probably inevitable. Daytime sleepiness could be either underestimated or overestimated. Second, there were relatively small samples. Had the numbers been larger, perhaps the results might have been more robust. Although there was a validation cohort, this was not a muticenter study, most importantly no external validation. The mean age of the series was very young (39–43 years old) with a BMI of 26.3 (clearly very thin). This is not the classical phenotype seen all over the world in sleep labs in patients with clinical suspicion of OSA except in China. Moreover, our samples were limited to Chinese patients. Therefore, future research with other ethnic groups is warranted to assess the generalisability of the current findings. Finally, residual confounding by several factors including habitual sleep duration, disorders not documented in the study, medications, and genetic and socioeconomic factors cannot be excluded [16].

## Conclusions

The weighted ESS orchestrated significant improvement in predicting the AHI, indicating that the capability in predicting the patients without OSAS or with severe OSAS was strong, which might have implications for clinical triage decisions to prioritize patients for polysomnography.

## Data Availability

The datasets used and/or analysed during the current study are available from the corresponding author on reasonable request.

## Abbreviations

OSA: Obstructive sleep apnoea
EDS: Excessive daytime sleepiness.
OSAS: Obstructive sleep apnoea syndrome
AHI: Apnea-hypopnea index
ESS: Epworth sleepiness scale
SD: Standard deviation
OR: Odds ratio
ROC: The receiver operating characteristic
AUC: The areas under the receiver operating characteristic curve
PLR: Positive likelihood ratio
NLR: Negative likelihood ratio
PPV: Positive predictive value
NPV: Negative predictive value
BMI: Body mass index
NC: Neck circumference
LOS: Lowest oxygen saturation
NDS: Normal daytime sleepiness
CI: Confidence interval
*r*_s_: Rank correlation coefficient.
